# Identification of critical residues at the C-terminal tip of ACKR4 regulating chemokine internalization and βarrestin involvement

**DOI:** 10.1186/s12964-024-01961-8

**Published:** 2024-12-02

**Authors:** Oliver J. Gerken, Nicola Catone, Daniel F. Legler

**Affiliations:** 1 Institute of Cell Biology and Immunology Thurgau (BITG) at the University of Konstanz, Kreuzlingen, CH-8280 Switzerland; 2 Graduate School for Cellular and Biomedical Sciences, University of Bern, Bern, CH-3012 Switzerland; 3 Theodor Kocher Institute, University of Bern, Bern, CH-3012 Switzerland; 4 Faculty of Biology, University of Konstanz, D-78464 Konstanz, Germany

**Keywords:** Chemokine, ACKR4, CCL19, Receptor tagging, βarrestin, PDZ proteins, Signaling

## Abstract

**Background:**

Atypical chemokine receptors (ACKRs) play an important role in regulating the availability of chemokines and are responsible for the formation of chemokine gradients required for the directed migration of immune cells in health and disease. ACKR4 shapes gradients of the chemokines CCL19 and CCL21, which are essential for guiding leukocyte homing to lymphoid organs where they initiate an adaptive immune response against invading pathogens. How ACKRs internalize and scavenge chemokines on the molecular level remains poorly understood. Current state-of the art methods to study βarrestin recruitment, signaling and trafficking of ACKRs - and G-protein-coupled receptors in general - rely heavily on C-terminally tagged receptors with unknown consequences for receptor functions.

**Methods:**

Fluorescently labelled CCL19 was used to quantify chemokine internalization by native and tagged receptors as assessed by flow cytometry and live cell confocal microscopy. Steady-state interaction and chemokine-driven recruitment of βarrestins was determined by NanoBiT bystander assays. βarrestin-dependency for CCL19 internalization was determined in wild-type versus βarrestin1/2-double deficient cell lines. Statistical significance was determined by unpaired t-test or one-way ANOVA with Dunnett’s or Tukey’s multiple comparison tests.

**Results:**

Addition of a C-terminal tag selectively affected the function of ACKR4, but not other ACKRs. Fusing a short peptide tag or a fluorescent protein to ACKR4 significantly augmented its ability to internalize its cognate ligand CCL19. In comparison to native ACKR4, its C-terminal tagging provoked an elevated pre-association of βarrestins with the plasma membrane, yet a reduction in chemokine-driven βarrestin recruitment. Furthermore, the addition of a C-terminal tag led to a shift from a βarrestin-dependent towards a βarrestin-independent endocytosis pathway. Similar results on chemokine uptake and on βarrestin-dependency were obtained with ACKR4 variants, in which a putative class II PDZ-binding domain located at the C-terminal tip of the receptor was mutated.

**Conclusion:**

This study identifies that the integrity of the C-terminus of ACKR4 is critical for receptor function. The addition of a C-terminal tag to ACKR4 enhances chemokine uptake and alters the involvement of βarrestins in receptor trafficking.

**Supplementary Information:**

The online version contains supplementary material available at 10.1186/s12964-024-01961-8.

## Background

Immune cell migration is a fundamental process of innate and adaptive immune responses governed by the recruitment and positioning of leukocytes. Guidance of immune cells is controlled largely by the tightly regulated local production of chemokines in tissues, by the restricted expression of cognate classical chemokine receptors on leukocytes, which transduce migratory signals [1, 2], and by atypical chemokine receptors (ACKRs), which are mainly expressed on barrier and stroma cells to remove excess of chemokines, limit their availability and shape chemotactic gradients [3, 4]. Chemokines are small, secreted proteins that induce directional cell migration and usually become immobilized in situ to provide guidance cues [2]. Leukocytes sense local chemokine gradients through classical chemokine receptors that span the plasma membrane sevenfold and couple to heterotrimeric small G-proteins to transmit signals required for directional migration [5, 6]. The activity and availability of chemokines is regulated by a small subfamily of atypical chemokine receptors (ACKRs). They are phylogenetically closely related and share the serpentine topology of classical chemokine receptors, but do not couple to G_i_-proteins and hence, do not elicit signals resulting in cell migration [7]. However, the four members of the ACKR subfamily (ACKR1-4) all have a high binding affinity for chemokines and control their bioavailability by scavenging, sequestration, or transport [3, 8].

ACKR4 is best known for its role in scavenging and shaping CCL19 and CCL21 gradients in lymph nodes to facilitate the homing of CC chemokine receptor 7 (CCR7)-expressing dendritic cells [9]. In fact, ACKR4 was shown to be expressed by lymphatic endothelial cells lining the ceiling of the subcapsular lymph node sinus, but not those lining the floor [9]. Moreover, the same study described a CCL21 gradient across the subcapsular sinus in wild-type, but not in ACKR4-deficient lymph nodes, which correlates with the CCL21 scavenging activity of ACKR4 and with CCL21-guided dendritic cell migration at these sites [9]. Besides CCL21, ACKR4 binds and internalizes the chemokines CCL19, CCL20, CCL22 and CCL25 [10, 11]. ACKR4 is also expressed on lymphatics, in the skin, the spleen, the thymus and the gut to promote leukocyte homing to secondary lymphoid organs and small intestine [12–15]. ACKR3 is crucial during embryogenesis, in neuronal and cardiovascular development controlling the migration of hematopoietic stem cells by scavenging the chemokine CXCL12 [16, 17]. The major function of ACKR2 is to scavenge inflammatory chemokines and sort them for degradation, thereby playing a critical role in the resolution of inflammatory responses [18]. ACKR1, the Duffy antigen receptor for chemokines, is predominantly expressed on erythrocytes and acts as sink and reservoir for chemokines in the blood [19]. Moreover, ACKR1 expressed on endothelial cells transports and presents numerous chemokines on the luminal site of venular vessels [20].

The knowledge on molecular mechanism(s) how ACKRs fulfil their scavenging function remains limited. However, with the exception of ACKR1, which transcytoses and presents chemokines and is largely present at the cell surface [20], all other ACKRs spontaneously traffic between the plasma membrane and endosomes. Upon chemokine binding, ACKRs internalize their cognate ligands and sort them for lysosomal degradation [3, 8]. The ability of ACKRs to scavenge chemokines has been linked to βarrestin1 and βarrestin2. In fact, βarrestins have consensually been shown to be recruited to chemokine stimulated ACKR2 [21, 22], ACKR3 [23, 24], and ACKR4 [25, 26]. Again, ACKR1 is an exception as this receptor is reported not to recruit and interact with βarrestins [27]. As for classical GPCRs, βarrestin recruitment to ACKRs depends on agonist-driven phosphorylation of serine/threonine residues situated at the receptor’s C-terminus by GPCR kinases (GRKs) or other protein kinases, resulting in clathrin-mediated receptor endocytosis [26, 28, 29]. Unexpectedly, in cells lacking βarrestins, ACKR2 [22], ACKR3 [24, 29] and ACKR4 [26, 30] are still able to take up chemokines. These findings indicate that βarrestins interact with these ACKRs and contribute to chemokine internalization, but are dispensable for chemokine scavenging. Notably, an ACKR3 mutant where all serine/threonine residues located at the receptor’s C-terminus were replaced by alanines (ACKR3 ST/A) failed to interact with GRKs and was also unable to internalize its ligand CXCL12, while wild-type ACKR3 in βarrestin-deficient cells still internalized the chemokine [29]. These data suggest that phosphorylation of ACKR3, but not βarrestins, regulates receptor trafficking and chemokine uptake. Moreover, knocking-in ACKR3 ST/A in mice rescued the lethal phenotype of systemic ACKR3 deficiency [29], indicating again that the C-terminus is critical for receptor function. For ACKR4, a truncated variant (ACKR4t) lacking the entire C-terminus failed to recruit GRKs and βarrestins in response to CCL19 stimulation and was unable to take up its ligand [26]. Furthermore, ACKR4t also lost its ability to interact with βarrestins under steady-state conditions [26]. Notably, the absence of βarrestins reduced (but did not completely block), whereas their overexpression enhanced CCL19 uptake by ACKR4 [26]. Moreover, attempts to express ACKR4 fused to GFP failed to yield transfectants expressing green fluorescence or functional receptor in one study [30], but was successful in other studies [10, 26]. Hence, it is tempting to speculate that the C-terminus of ACKR4, and potentially other ACKRs, might comprise regulatory elements controlling receptor expression, trafficking and function. PDZ-domain containing proteins could be potential regulators of ACKRs as such proteins have been show to interact with canonical GPCRs and ion channels to modulate their functions [31, 32]. Notably, current state-of the art methods to measure the interaction or the recruitment of signaling proteins to ACKRs (and GPCRs), as well as to assess receptor trafficking depend largely on nanoluciferase and/or BRET-based assays [33, 34]. The caveat of these assays is that they rely heavily on C-terminally tagged receptors and hence, the introduced fluorophores or enzymes might jeopardize the regulatory function of the C-terminus and/or mask the regulatory domain(s) located at the tail of the receptor. This prompted us to undertake a comprehensive and detailed investigation into the function of the native C-terminus of ACKR4 in receptor trafficking and in chemokine uptake, and to compare native ACKR4 with tagged receptor variants.

## Methods

### Cloning of expression plasmids

Expression plasmids were generated by cloning PCR amplified inserts using the primer pairs listed in Tables S1-S3, followed by restriction enzyme digestion and ligation into pcDNA3, pIRES or pSUMO backbones. Single- or double-point mutations of ACKR4 were introduced by site-directed mutagenesis with oligonucleotides listed in Table S4. Reagents for cloning were purchased from ThermoFisher (Waltham, MA, USA). All vectors were verified for correct cloning by sequencing (Microsynth AG, Balgach, Switzerland).

### Cell culture and transient transfection

HEK293 parental and HEK293 βarrestin1/2^−/−^ cells were kindly provided by Dr. Stephane Laporte [35]. HEK293 cells and HeLa cells were maintained in DMEM (Capricorn Scientific GmbH, Ebsdorfergrund, Germany) supplemented with 10% FCS (Lonza, Basel, Switzerland) and 1% penicillin-streptomycin (Pan Biotech, Aidenbach, Switzerland) at 37 °C, 5% CO_2_ and 95% humidity. HEK293 and HeLa cells do not endogenously express ACKR4.

Cells were transiently transfected using the Neon Transfection System (ThermoFisher, Waltham, MA, USA) according to the manufacturer protocol. Briefly, 5 × 10^5^ cells were mixed with 5 µg plasmid DNA and electroporated at 1100 V, two pulses with a width of 20 ms (HEK293 cells) or at 1005 V, two pulses with a width of 35 ms (HeLa cells). Plasmid ratio between receptors, lgBiT-CAAX and smBiT-βarrestins were 5:2:1, respectively. Post transfection, cells were cultivated for 24 h in DMEM containing 20% FCS before experiments were conducted. Transfection efficiency was > 95% for HEK293 and > 70% for HeLa cells.

### Chemokine production

All human chemokines (CCL19, CXCL12, CCL5 E66S and CCL19-S6) were expressed in the *E.coli* strain BL21(DE3) and purified as previously described [36]. Subsequent site-specific CCL19-S6 labelling with Dy649P1 (Dyomics GmbH, Jena, Germany) and pHRodo (ThermoFisher, Waltham, MA, USA) was performed as published [36–38]. For human CCL5, a single-point mutation (E66S) was introduced to stabilize the protein during purification and to prevent its precipitation [39].

### Flow cytometry

For receptor surface staining, transiently transfected cells were stained with a primary anti-ACKR4 antibody (Sigma-Aldrich, Buchs, Switzerland; #362102, dilution 1:750) on ice for 30 min followed by intensive washing and incubation with a secondary anti-mouse antibody coupled to AlexaFluor^647^ (ThermoFisher; #A-21235, dilution 1:1000). Receptor transfected HeLa cells were incubated at 37 °C up to 45 min with 25 nM CCL19-S6^Dy649P1^ and up to 60 min with 25 nM CCL19-S6^pHRodo^ to measure chemokine uptake. Chemokine uptake studies in transfected HEK293 cells were performed using 5 nM CCL19-S6^Dy649P1^ at 37 °C for up to 45 min. Chemokine uptake experiments were conducted in a Hepes buffered solution containing 10 mM Hepes, 145 mM NaCl, 5 mM KCl, 1 mM MgCl_2_, 1 mM CaCl_2_, 1 mM Na_2_HPO_4_ and 5 mM glucose, and cell associated fluorescence (mean fluorescence intensities; MFIs) were measured on a BD LSRFortessa flow cytometer using the BD FACSDiva™ 9 software (BD Biosciences, San Jose, CA, USA). Data were analyzed with the FlowJo software (BD Biosciences, San Jose, CA, USA).

### Bystander NanoBiT assay

Receptor transfected HEK293 cells were washed and resuspended in PBS supplemented with 5 mM glucose, and distributed in duplicates to a 96-well half-well plate (PerkinElmer, Waltham, MA, USA). 5 µM coelenterazine H (Biosynth, Staad, Switzerland) was added to the cells and incubated for 5 min at 37 °C. Baseline luminescence was measured every 30 s (385–440 nm, 350 ms integration time) for further 5 min on a Tecan Spark 10 M multiplate reader (Tecan, Männdorf, Switzerland), followed by stimulation with the respective chemokines or PBS. If not specified otherwise, 1 µM of chemokine was used to reach maximal responses. The emitted luminescence was monitored every 30 s for additional 15 min. For analysis of the recorded data, values of the wells comprising stimulated cells were divided by values of wells with the unstimulated ones (ΔRLU). Subsequently, the area under the curve (AUC) was determined for quantification [40].

### Live fluorescent confocal microscopy

Receptor expressing cells were seeded in 35 mm imaging dishes (Ibidi, Gräfelfing, Germany) for live cell imaging one day after transient transfection. Cells were extensively washed and kept in Hepes buffered solution (see section *flow cytometry*) and analyzed on a Leica TCS SP5 II confocal microscope (Leica, Wetzlar, Germany) equipped with an Argon (488 nm), HeNe (543 nm) and HeNe (633 nm) excitation laser that was preheated to 37 °C. Images were acquired every 32 s sequentially with new frames for YPet (514–544 nm), mScarlet (578–608 nm) and CCL19-S6^Dy649P1^ (656–686 nm) using the HyD detector. After the measurement of 6 frames serving as baseline, 10 nM CCL19-S6^Dy649P1^ was added and cells were imaged for additional 91 frames (total of 52 min). For quantification, the corrected total cell fluorescence (CTCF) of the cells was calculated, and in a second step normalized to the maximum value measured per experiment using the Fiji-based ImageJ software.

### Statistical analysis

Statistical analysis was performed using ordinary one-way ANOVA with Dunnett’s or Tukey’s multiple comparison test and single pooled variance or unpaired t-test for the comparison of two samples (GraphPad Prism Software v10.1.2, San Diego, CA. USA). * *p* < 0.05; ** *p* < 0.01; *** *p* < 0.001; **** *p* < 0.0001.

## Results

### C-terminal tagging of ACKR4 enhances its capacity to internalize CCL19

**Fig. 1:**
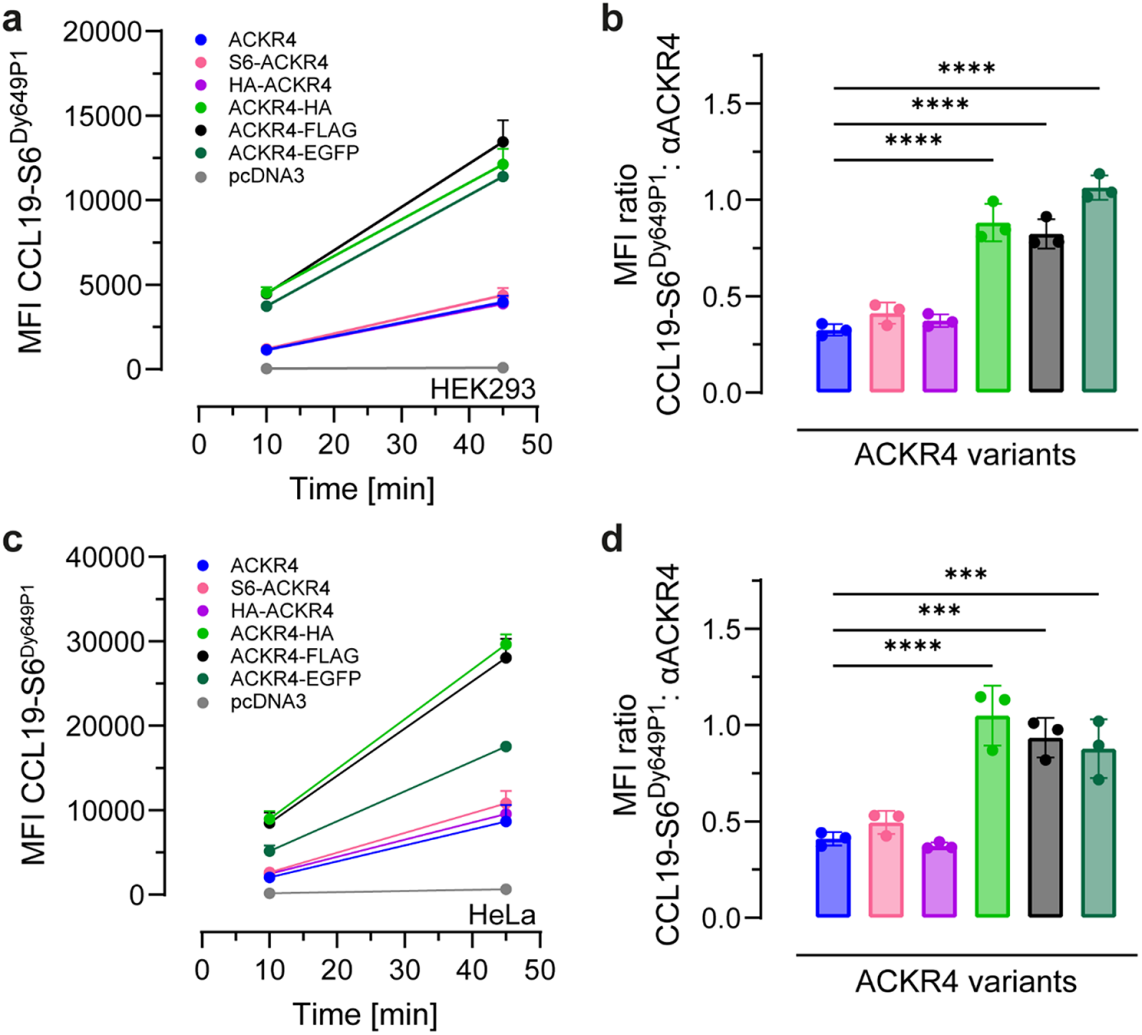
Addition of C-terminal tags to ACKR4 enhances chemokine internalization. **a** Augmented CCL19 endocytosis in HEK293 cells expressing ACKR4 with a C-terminal tag. HEK293 cells transiently expressing native and tagged ACKR4 variants were incubated at 37 °C for 10 and 45 min with 5 nM CCL19-S6^Dy649P1^ and chemokine-derived fluorescence (MFI) determined by flow cytometry. Transfection with empty vector (pcDNA3) served as negative control. *n* = 3, mean ± SD. **b** MFI ratio between CCL19-S6^Dy649P1^ uptake (at t = 45 min determined in **a**) and receptor surface expression of the different ACKR4 variants in HEK293 cells. *n* = 3, mean ± SD. **c** Enhanced CCL19 internalization in HeLa cells expressing ACKR4 with a C-terminal tag. HeLa cells transiently expressing native and tagged ACKR4 variants were incubated at 37 °C for 10 and 45 min with 25 nM CCL19-S6^Dy649P1^ and chemokine-derived fluorescence (MFI) determined by flow cytometry. *n* = 3, mean ± SD. **d** MFI ratio between CCL19-S6^Dy649P1^ uptake (at 45 min determined in **c**) and receptor surface expression of indicated ACKR4 variants in HeLa cells. *n* = 3, mean ± SD. Statistical analysis: ordinary one-way ANOVA, *** *p* < 0.001, **** *p* < 0.0001

Discrepant results on the ability to functionally express ACKR4 fused to fluorescent proteins have been reported in the literature [10, 26, 30]. Unexpectedly, in pilot experiments we noted substantial differences in the ability to internalize CCL19 between cell transfectants expressing various ACKR4 constructs. This prompted us to systematically assess the influence of tagging ACKR4 on its function. We cloned human native, untagged ACKR4, as well as C-terminal and N-terminal variants thereof into the common expression vector pcDNA3. We have chosen to fuse the fluorophore EGFP, which is routinely used as intracellular fusion protein of transmembrane proteins, to the C-terminus of ACKR4. In addition, we fused the S6-peptide, commonly used for cell surface labelling, to the N-terminus of ACKR4. Moreover, we used two short peptides, namely the HA-tag and the FLAG-tag, which are frequently used for Western blotting, and fused it to the C- and/or N-terminus of ACKR4. HEK293 cells transiently expressing the different ACKR4 variants were incubated for 10 and 45 min with 5nM of site-specifically fluorescent-labelled CCL19-S6^Dy649P1^ at 37 °C to allow chemokine binding and uptake, which was measured and quantified by flow cytometry. All cell lines expressing one of the ACKR4 variants readily took up CCL19-S6^Dy649P1^ and cell associated fluorescence accumulated over time (Fig. 1a). Interestingly, CCL19-S6^Dy649P1^ uptake was about three times higher in cells expressing C-terminally tagged ACKR4 as compared to untagged, native ACKR4 (Fig. 1a). N-terminally tagged ACKR4 behaved like untagged receptor (Fig. 1a). The enhanced CCL19-S6^Dy649P1^ uptake by C-terminally tagged ACKR4 was not due to differences in transfection efficiency, as more than 95% of all cells were positive for ACKR4 among all receptor variants, or due to increased surface expression of the corresponding receptor variants (Figure S1a-c). Normalizing the capacity of chemokine uptake by calculating the ratio between the fluorescence derived from CCL19-S6^Dy649P1^ and the fluorescence from antibody-mediated surface staining revealed that C-terminal tagging of ACKR4, with either a small peptide or a fluorophore, significantly enhanced the receptor’s ability to take up the chemokine (Fig. 1b). To corroborate this finding, we determined chemokine internalization by the ACKR4 variants also in HeLa cells. Similarly to HEK293 cells, CCL19-S6^Dy649P1^ uptake was 2 to 3-fold higher in HeLa cells expressing C-terminally tagged ACKR4 variants compared to native or N-terminally tagged receptor variants (Fig. 1c). Receptor surface staining at 4 °C served as control for expression and normalization in HeLa cells (Fig. 1d, Figure S1d-f). Hence, in both cell types, fusing a short peptide tag or a fluorescent protein to the C-terminus of ACKR4 significantly enhanced its capacity to internalize chemokines.

**Fig. 2:**
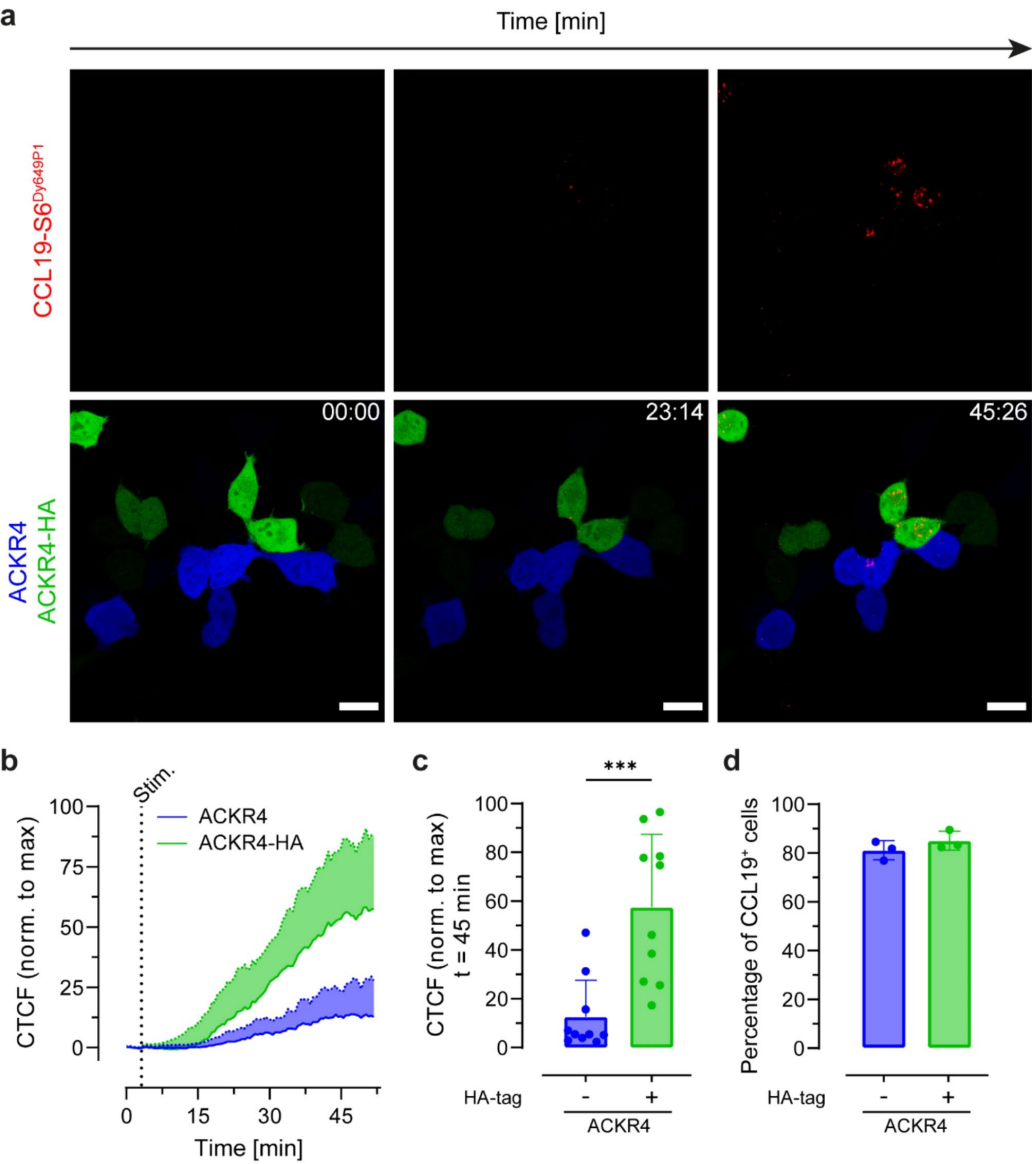
Live cell confocal imaging reveals earlier and more efficient chemokine internalization by ACKR4-HA compared to native receptor. **a** Serial confocal images of mixed HEK293 cell populations transiently transfected with either pIRES mScarlet_ACKR4 (blue) or YPet_ACKR4-HA (green) that were stimulated with 10 nM CCL19-S6^Dy649P1^ (red). Representative images derived from three independent experiments. Scale bar: 20 μm. **b** Corrected total cell fluorescence (CTCF) derived from internalized CCL19-S6^Dy649P1^ was normalized to the maximal measured intensity in (**a**). **c** Quantification of measured CTCF from (**a**) at time point t = 45 min; *n* = 3, total of 20 cells, mean ± SD. **d** Percentage of ACKR4 or ACKR4-HA transfected cells internalizing CCL19-S6^Dy649P1^. *n* = 3 independent experiments, mean ± SD. Statistical analysis: unpaired t-test, *** *p* < 0.001

Next, we examined the rates of chemokine internalization by native ACKR4 and ACKR4-HA in a competitive live cell imaging setup. To this end, HEK293 cells were transiently, but separately transfected with an IRES vector coding for mScarlet and native ACKR4, or for YPet and ACKR4-HA. The two transfected cell lines were mixed in a 1:1 ratio, seeded in the same imaging chamber, stimulated with 10 nM of CCL19-S6^Dy649P1^, and fluorescence was recorded over time by confocal live cell imaging. Cells expressing YPet and ACKR4-HA (represented in green) showed more efficient CCL19-S6^Dy649P1^ uptake (shown in red) than cells expressing mScarlet and ACKR4 (depicted in blue) (Fig. 2a). To quantitatively assess and compare chemokine internalization over time by ACKR4 and ACKR4-HA, the cell with the highest total fluorescence (maximum) derived from CCL19-S6^Dy649P1^ at the end of the recording time of one experiment was identified and used as reference. The total (chemokine) fluorescence for each cell over time was corrected and normalized to the reference. Quantitative assessment of CCL19-S6^Dy649P1^ internalization over time by cells expressing ACKR4 (and mScarlet) or ACKR4-HA (and YPet) revealed that C-terminal tagging of ACKR4 accelerated and enhanced its capacity to take up CCL19 (Fig. 2b). In fact, our data demonstrate that, over a period of 45 min, cells expressing ACKR4-HA are on average 4.5-times more efficient in internalizing the chemokine than cells expressing native ACKR4 (Fig. 2c), while the percentage of cells internalizing the chemokine was comparable for cells expressing ACKR4-HA or its untagged counterpart (Fig. 2d). Hence, fusing a C-terminal tag to ACKR4 profoundly influences its capacity to internalize its cognate chemokine ligand.

### C-terminal tagging selectively affects βarrestin recruitment to ACKR4 with no discernible impact on other ACKRs

**Fig. 3:**
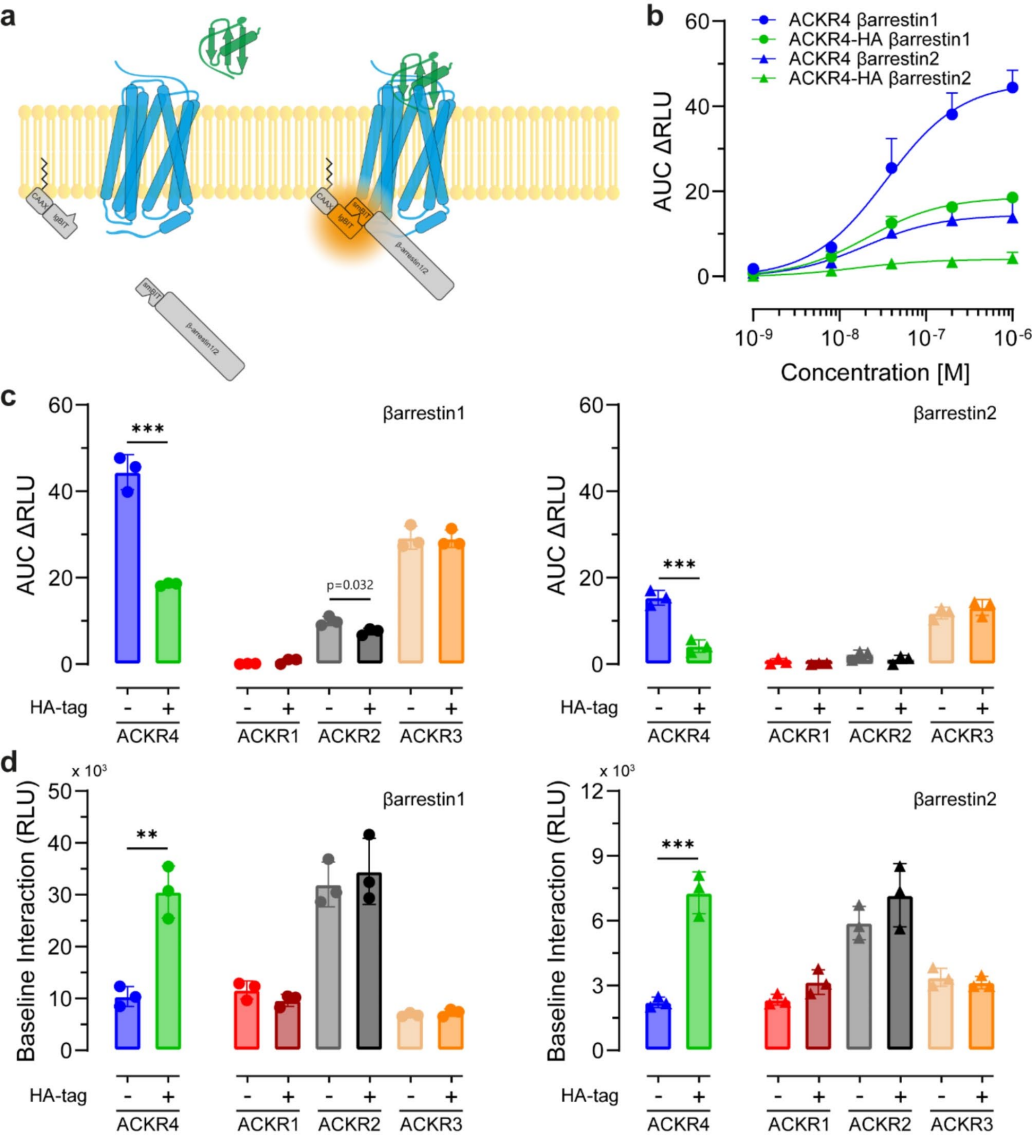
Fusion of a C-terminal HA-tag selectively affects βarrestin interaction and recruitment of ACKR4, but not other ACKRs. **a** Scheme of the bystander NanoBiT assay used to measure the recruitment of βarrestins to the plasma membrane upon stimulation of a receptor with its ligand. **b** CCL19 concentration dependent recruitment of βarrestins upon stimulation of native and C-terminal tagged ACKR4. Quantification (AUC) of HEK293 cells transiently transfected with ACKR4 or ACKR4-HA, together with lgBiT-CAAX and smBiT-βarrestin1 or smBiT-βarrestin2 upon stimulation with graded concentrations of CCL19; *n* = 3, mean ± SD. **c** Comparison of chemokine-driven recruitment of βarrestin1 (left) and βarrestin2 (right) to the plasma membrane of HEK293 cells transiently expressing native and C-terminally HA-tagged ACKRs. CCL5 (1µM) was used to stimulate ACKR1(-HA) and ACKR2(-HA), CXCL12 (1µM) for ACKR3(-HA) and CCL19 (1µM) for ACKR4. *n* = 3, mean ± SD. **d** Pre-association of βarrestin1 (left) and βarrestin2 (right) with the plasma membrane (i.e. CAAX) of ACKR expressing HEK293 cells. Basel interaction in the absence of chemokine stimulation is depicted. *n* = 3, mean ± SD. Statistical analysis: unpaired t-test, ** *p* < 0.01; *** *p* < 0.001

Next, we tested whether C-terminal tagging of ACKR4 also affects chemokine-driven βarrestin recruitment. To investigate this, we established a bystander NanoBiT assay in which we fused the larger fragment of the split-nanoluciferase (referred to as lgBiT) to the plasma membrane anchor CAAX and the small peptide fragment of the split-nanoluciferase (smBiT) to βarrestins. The co-transfection of the lgBiT and smBiT fusion molecules together with a receptor enables a side-by-side comparison of βarrestin recruitment to the plasma membrane upon ligand stimulation of either native or tagged receptors (Fig. 3a). We found that stimulation of native ACKR4 with graded concentrations of CCL19 efficiently recruited βarrestins to the plasma membrane with a preference for βarrestin1 over βarrestin2 (Fig. 3b). Our data are in line with previous results using a direct NanoBiT assay, where βarrestins are recruited directly to the receptor [11]. Notably, CCL19-driven βarrestin recruitment to the plasma membrane was profoundly reduced if ACKR4 was C-terminally tagged (Fig. 3b). Indeed, the fusion of a C-terminal HA-tag to ACKR4 resulted in a reduction in CCL19-driven βarrestin1 recruitment to 42% and to 27% for βarrestin2 as compared to native ACKR4 (Fig. 3b).

To investigate whether tagging also affects other ACKRs, we C-terminally fused an HA-tag to all other ACKRs. We stimulated cells expressing ACKR1(-HA) and ACKR2(-HA) with 1 µM CCL5, and cells transfected with ACKR3(-HA) with 1 µM CXCL12 to trigger a maximal response. For comparison, we also included ACKR4(-HA) expressing cells that were also stimulated with 1 µM CCL19 (Fig. 3c). Consistent with a previous report [27], ACKR1 did not recruit βarrestins in response to CCL5, regardless of the presence or absence of an HA-tag (Fig. 3c). Stimulation of ACKR2 expressing cells with CCL5 resulted in the recruitment of βarrestin1 to the plasma membrane, and fusing an HA-tag to its C-terminus had little effect on βarrestin1 recruitment (Fig. 3c). Stimulation of ACKR3 with CXCL12 led to a robust and comparable βarrestin recruitment to the plasma membrane, independent of whether the receptor was C-terminally tagged or not (Fig. 3c).

In addition, we determined whether the expression of ACKRs, in the absence of cognate ligand engagement, modulate βarrestin association to the plasma membrane. Surprisingly, expression of ACKR4-HA resulted in a profound pre-association of both βarrestins with the plasma membrane in the absence of CCL19, that was 2.9 (for βarrestin1) and 3.3 (for βarrestin2) times higher as compared to cells expressing native ACKR4 (Fig. 3d). We also noted differences in the plasma membrane association of βarrestins in cells expressing ACKR1-3 under steady-state conditions. However, basal interactions of βarrestins with the plasma membrane of cells expressing ACKR1-3 was observed regardless of the presence or absence of a C-terminal HA tag (Fig. 3d).

Hence, the addition of a C-terminal tag selectively affects ACKR4 with no discernible impact on any other ACKR. In fact, C-terminal tagging of ACKR4 led to an increase in βarrestin pre-association with the receptor at the plasma membrane. This suggests a higher receptor cycling rate in the absence of ligands, and is likely to explain the elevated endocytosis of CCL19 by C-terminally tagged ACKR4.

### C-terminal tagging of ACKR4 affects efficiency and βarrestin-dependency for CCL19 endocytosis

**Fig. 4:**
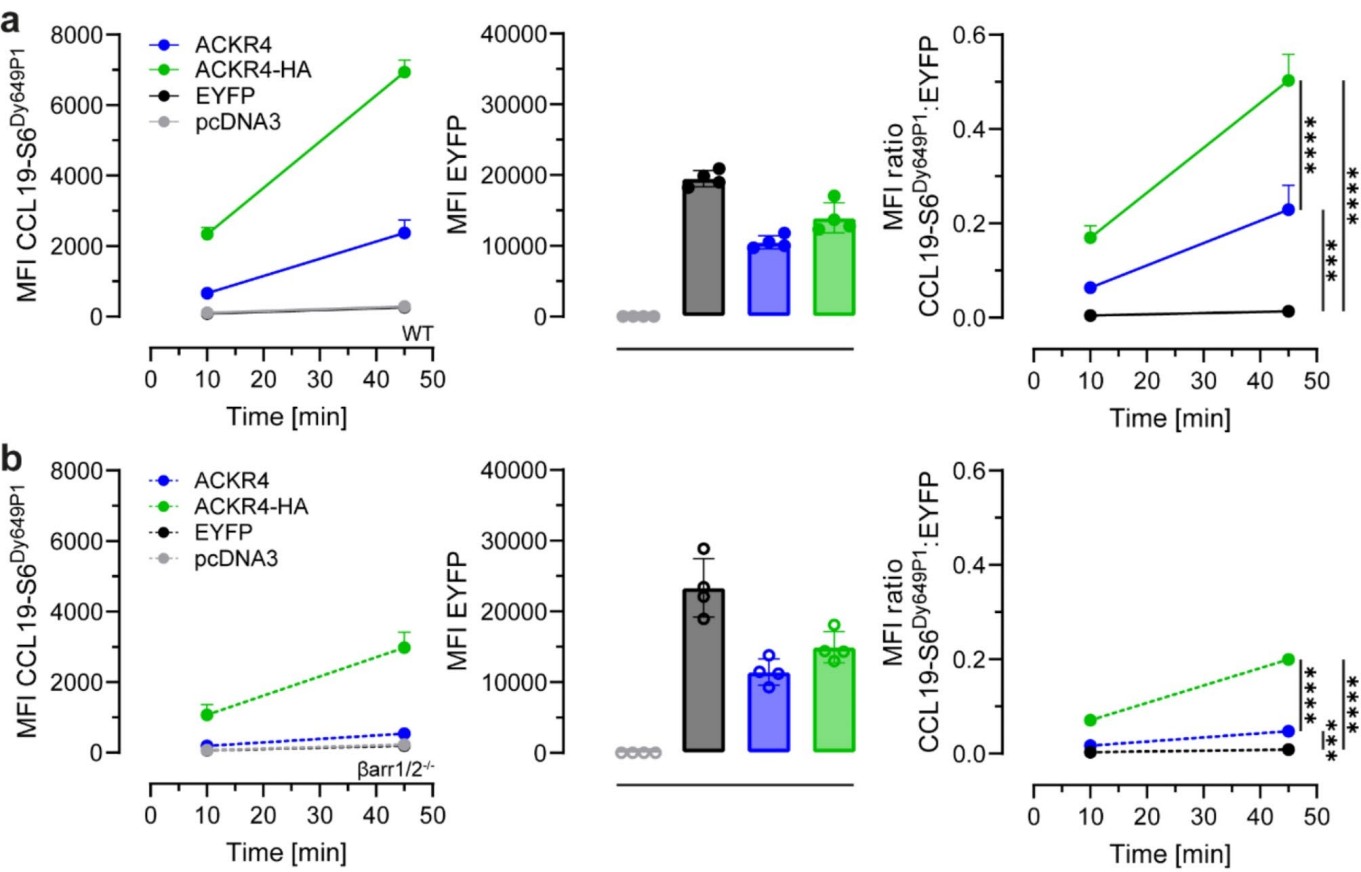
Addition of a C-terminal tag to ACKR4 affects efficiency and βarrestin dependency of CCL19 internalization. HEK293 wild-type (WT; **a**) or βarrestin1/2^-/-^ (βarr1/2^-/-^; **b**) cells were transiently transfected with an empty vector, EYFP only or pIRES vectors encoding for EYFP together with either ACKR4 or ACKR4-HA, stimulated with 5 nM CCL19-S6^Dy649P1^ and analyzed by flow cytometry. Chemokine internalization (left), EYFP expression (middle) and MFI ratio between CCL19-S6^Dy649P1^ uptake and EYFP (right) is shown. *n* = 4, mean ± SD. Statistical analysis: ordinary one-way ANOVA of t = 45 min, ** *p* < 0.01; **** *p* < 0.0001

As C-terminal tagging of ACKR4 perturbs steady-state interaction and chemokine-driven βarrestin recruitment, as well as CCL19 uptake, we wondered whether this also affects the dependency of ACKR4 on βarrestins for chemokine scavenging. To address this in a controlled manner, we transfected (parental) HEK293 cells with pIRES vectors encoding for EYFP and native ACKR4, or EYFP and ACKR4-HA, as well as an empty vector and a vector coding for EYFP as controls and incubated them with 5 nM CCL19-S6^Dy649P1^. This allowed us to monitor and quantify chemokine uptake and receptor expression by flow cytometry in a single experiment. Consistent with results shown in Fig. 1, ACKR4-HA displayed a 2.2-times higher CCL19-S6^Dy649P1^ internalization than untagged ACKR4 at comparable receptor expression levels (Fig. 4a). Normalization of chemokine uptake by receptor expression revealed a clearly enhanced endocytosis ability of ACKR4-HA over ACKR4 in internalizing CCL19 (Fig. 4a). Next, we investigated chemokine uptake by ACKR4 in HEK293 lacking both βarrestins (βarr1/2^−/−^). Although reduced, βarrestin-deficient cells were still able to internalize CCL19-S6^Dy649P1^ by ACKR4-HA (Fig. 4b). In marked contrast, βarrestin-deficient cells failed to endocytose CCL19-S6^Dy649P1^ by native, untagged ACKR4 (Fig. 4b). Thus, C-terminal tagging of ACKR4 not only affects steady-state and ligand-induced interaction with βarrestins, it equally affects the potency and dependency for βarrestins in taking up CCL19.

### Mutating two hydrophobic residues at the C-terminal tip representing a putative class II PDZ-binding domain recapitulates the phenotype of C-terminally tagged ACKR4

**Fig. 5:**
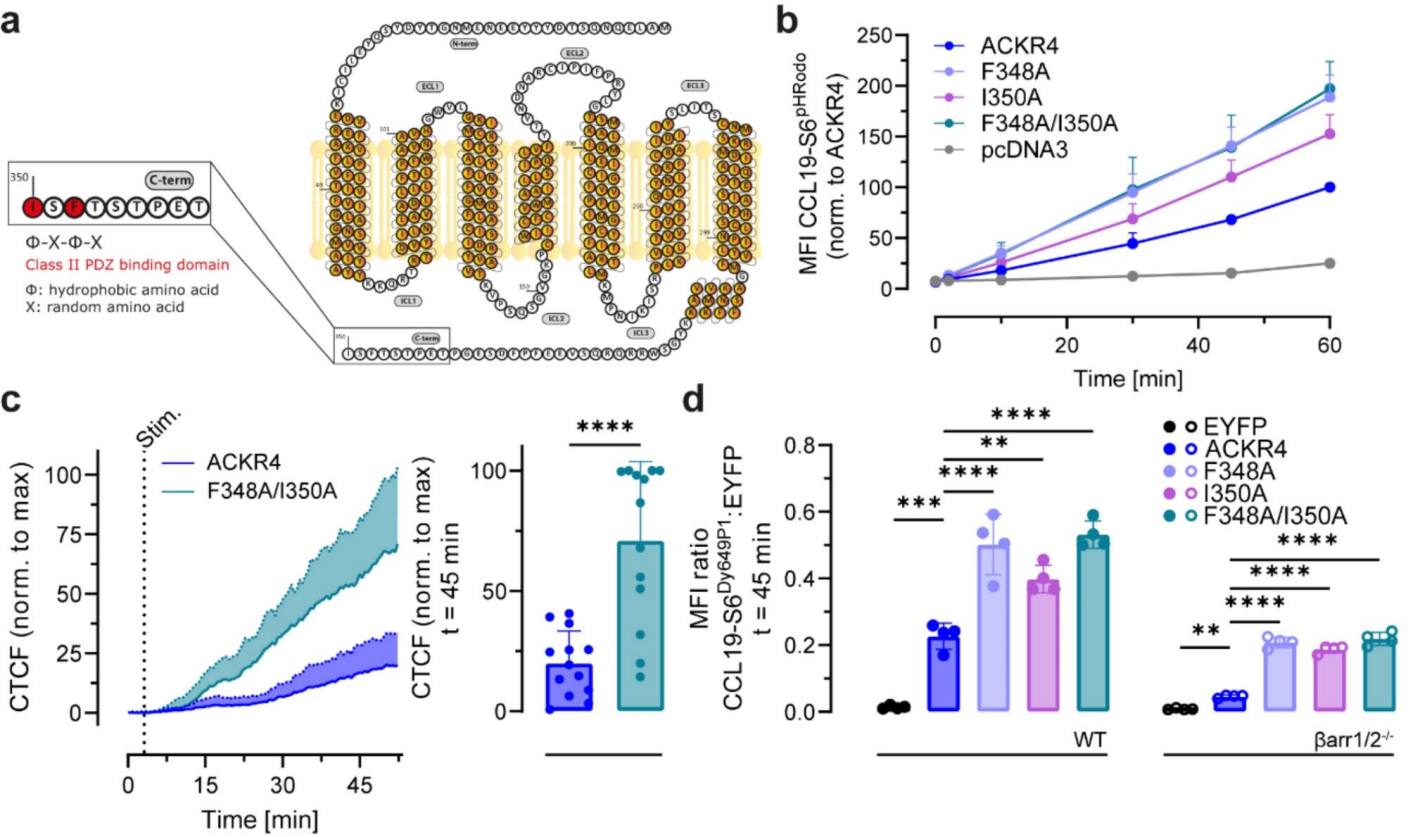
Mutation of a putative class II PDZ-binding domain at the tip of the tail recapitulates the phenotype of ACKR4-HA. **a** Illustration of the secondary structure of ACKR4 including a putative class II PDZ-binding domain (red). **b** CCL19 internalization by ACKR4 mutants. HeLa cells transiently expressing ACKR4 and point mutants thereof were incubated with 25 nM CCL19-S6^pHRodo^, chemokine derived fluorescence was determined by flow cytometry and normalized to the maximum uptake by wild-type ACKR4. Transfection with pcDNA3 empty vector served as negative control. *n* = 3, mean ± SD. **c** Mixed HEK293 cell populations transiently transfected with either pIRES mScarlet_ACKR4 or YPet_ACKR4 F348A/I350A were stimulated with 10 nM CCL19-S6^Dy649P1^ and analyzed by confocal microscopy. Corrected total cell fluorescence (CTCF) derived from internalized CCL19-S6^Dy649P1^ was normalized to the maximal measured intensity reached at time point t = 45 min. *n* = 3, total of 26 cells, mean ± SD. **d** Wild-type (WT, left) and βarrestin1/2^-/-^ (βarr1/2^-/-^, right) HEK293 cells were transiently transfected with an empty vector, EYFP only or pIRES vectors encoding for EYFP together with indicated ACKR4 variants, stimulated with 5 nM CCL19-S6^Dy649P1^ for 45 min and analyzed by flow cytometry. The MFI ratio between internalized CCL19-S6^Dy649P1^ and EYFP for each receptor variant is shown. *n* = 4, mean ± SD. Statistical analysis: ordinary one-way ANOVA, ** *p* < 0.01; **** *p* < 0.0001

So far, we have discovered that the addition of a C-terminal tag to ACKR4 has a major impact on ligand endocytosis and interaction with βarrestins, but we lack information as to why this is so. By thoroughly analyzing the amino acid sequence of the receptor tail, we identified a putative class II PDZ-binding domain at the C-terminal tip of ACKR4 (Fig. 5a). In fact, the last four amino acids of ACKR4, namely -T-F-S-I, match the characteristics of the consensus sequence of a class II PDZ-binding domain, which is -X-Φ-X-Φ_COO_^−^, where X represents a random and Φ a hydrophobic amino acid [41]. Consequently, we generated receptor mutants in which F348 or I350 were exchanged for alanine, either alone or in combination. To test whether these residues of ACKR4 are important for chemokine endocytosis, we stimulated transiently transfected cells with 25 nM CCL19-S6^pHRodo^, which becomes fluorescent only in an acidic environment, such as early endosomes or lysosomes. Interestingly, cells expressing the ACKR4 mutants F348A and F348A/I350A showed highest CCL19-S6^pHRodo^ uptake, whereas the ACKR4 mutant I350A showed moderate increase as compared to wild-type ACKR4 (Fig. 5b). In our competitive live cell imaging setup with mixed cell populations expressing either mScarlet and ACKR4 or YPet and ACKR4 F348A/I350A revealed that the ACKR4 mutant F348A/I350A was faster and significantly more efficient in internalizing CCL19-S6^Dy649P1^ than wild-type ACKR4 (Fig. 5c), analogous to ACKR4-HA and native ACKR4 (Fig. 2). Moreover, we compared CCL19-S6^Dy649P1^ uptake in parental and βarrestin1/2^−/−^ HEK293 cells and found that, although reduced, βarrestin-deficient cells were able to internalize CCL19-S6^Dy649P1^ by the ACKR4 mutants F348A and F348A/I350A (as by ACKR4-HA), but not by wild-type (native) ACKR4 (Fig. 5d and Figure S2a-b).

In summary, we uncovered a critical role of a free C-terminus for ACKR4 trafficking and function. In fact, fusing a small peptide tag or a fluorescent protein to the C-terminus of ACKR4 increases the steady-state interaction with βarrestins, while reducing chemokine-driven recruitment of βarrestins to the receptor, resulting in enhanced chemokine internalization. Moreover, introducing point mutations in the consensus sequence for a putative class II PDZ-binding domain located at the C-terminal tip of ACKR4 recapitulates the effects seen for C-terminally tagged receptors in terms of chemokine internalization and βarrestin involvement. Our data provide evidence that ACKR4 includes a class II PDZ-binding domain at its tail and that a free C-terminus is critical for regulating ACKR4 functions, but not for other ACKRs.

## Discussion

The knowledge on molecular mechanism(s) of how ACKR4 and ACKRs in general fulfil their scavenging function remains limited. Current state-of the art methods to study βarrestin interaction and agonist-driven recruitment, as well as trafficking of ACKRs rely predominantly on C-terminally tagged receptors enabling BRET or NanoBiT measurements or their visualization by fluorescence microscopy. Potential consequences of adding tags to receptors are altered properties and functions that are often neglected due to lack of appropriate alternatives or tools.

In the present study, we uncovered that the integrity of the C-terminus of ACKR4 is critical for its function. In fact, the addition of a C-terminal tag to ACKR4 enhanced chemokine uptake and altered the involvement of βarrestins in receptor trafficking. Previous studies using C-terminally tagged ACKR4 described that βarrestins are dispensable for chemokine uptake and scavenging [26, 30]. Remarkably, as alternative trafficking route for ACKR4, internalization by caveolae has already been proposed [30]. Here, we present data showing that omitting the tag at the C-terminus of ACKR4 shifted chemokine endocytosis from a βarrestin-independent to a βarrestin-dependent mechanism. Notably, the chemokine receptor CCR1 (but not CCR2) was shown to have different properties upon addition of a C-terminal tag. Compared to native receptor, CCR1 fused to the luciferase RLucII remained trapped in intracellular compartments and did not reach the cell surface [42]. In line with a previous report [26], we here found no evidence of tagged ACKR4 to be retained intracellularly, but more likely ACKR4-HA cycles faster between the plasma membrane and endosomal compartments affecting steady-state trafficking and the chemokine scavenging function.

By inspecting the amino acid sequence of the ACKR4 tail, we noted that the last four amino acids, which are T-F-S-I, match the characteristics of the consensus sequence (X-Φ-X-Φ_COO_^−^) of a class II PDZ-binding domain [41]. By contrast, no such consensus sequence is present at the tip of the tail in ACKR1, ACKR2 and ACKR3. Single- and double-point mutation of the hydrophobic amino acids (Φ) F348 and I350 of ACKR4 to alanine resulted in faster and augmented ability to internalize CCL19, and rendered chemokine uptake independent of βarrestins. Collectively, our data indicate that the tip of the tail of ACKR4 includes a class II PDZ-binding domain that controls receptor functions. The addition of a C-terminal tag to ACKR4 may shield this regulatory domain and renders the receptor more active. Further studies are mandatory to substantiate our findings. However, there are increasing numbers of studies reporting on the involvement of PDZ proteins in GPCR trafficking and signaling. For instance, endocytosis of the β1-adrenergic receptor decreased upon interaction with PSD95 [43], whereas the interaction of NHERF1 with CCR5 increased receptor endocytosis [44]. Additionally, signaling of CCR5 through ERK, FAK, and Rho was reported to increase in the presence of NHERF1 [44, 45]. Hence, PDZ proteins appear to be involved in various regulatory processes and control signaling and trafficking of GPCRs in either an enhancing or an attenuating manner.

PDZ proteins can bind their targets in two different ways, either through an internal or a C-terminal domain. The binding groove of a PDZ protein usually consists of the β2-strand, the α2-helix and is capped at one end by the carboxylate-binding loop [32]. An internal interaction involves a PDZ protein with a distinct binding pocket allowing the alignment of the PDZ-binding domain into this binding groove and a hairpin or a bend in the structure of the ligand to prevent structural hindrance with the carboxylate-binding loop. For example, PAR6 binds its ligand PALS1 through an internal interaction, which is enabled by a bend in PALS1, and the PDZ protein nNOS, which creates an intramolecular interaction with the PDZ protein α1-syntrophin via a hairpin structure [46, 47]. The more common binding mode represents the C-terminal interaction of the PDZ protein and its ligand. Here, the amino acids of the ligand bind to the binding groove of the PDZ protein, whereas the last amino acid in the C-terminus of the ligand interacts with the carboxylate-binding loop, conjointly stabilizing the PDZ-ligand complex as reported for the binding of PSD95 to its ligand CRIPT [32, 48]. Thus, the possible involvement of PDZ proteins in ACKR4 trafficking and function could be explained by the necessity of the C-terminal residues not only to mediate the interaction with a putative PDZ protein, but also to stabilize the PDZ-ligand complex, which is disrupted by mutation of the hydrophobic residues responsible for the interaction with the binding groove and by the addition of a C-terminal tag that creates a structural hindrance with the PDZ protein and its carboxylate binding loop.

## Conclusion

This study identifies that the integrity of the C-terminus of ACKR4 is crucial for receptor function. The addition of a C-terminal tag to ACKR4 enhances its ability to internalize chemokines. We provide evidence that fusing a C-terminal tag to ACKR4 results in pre-association of βarrestins in the absence of its ligand. Chemokine stimulation of tagged ACKR4, through pre-coupling with βarrestins, enables faster and more efficient chemokine internalization. This seems to be an ACKR4 specific property, as tagging other ACKRs did not show this effect. Moreover, this study identifies a putative class II PDZ-binding domain that may serve as additional regulatory mechanism for chemokine scavenging by ACKR4.

## Electronic supplementary material

Below is the link to the electronic supplementary material.

Supplementary Material 1

## Data Availability

The datasets supporting the conclusions of this article are available on Zenodo, 10.5281/zenodo.13926103.

## Abbreviations

ACKR: Atypical chemokine receptor
AUC: Area under the curve
CCR: CC chemokine receptor
CTCF: Corrected total cell fluorescence
DC: Dendritic cell
GPCR: G protein-coupled receptor
MFI: Mean fluorescence intensity
RLU: Relative luminescence units
