# Supplementary material for: Identification of critical residues at the C-terminal tip of ACKR4 regulating chemokine internalization and βarrestin involvement
Source: Cell Commun Signal. 2024 Dec 2;22:576. doi: 10.1186/s12964-024-01961-8 (PMC11610291; doi:10.1186/s12964-024-01961-8)
Supplement: Supplementary file 1 — Supplementary Material 1 [file 12964_2024_1961_MOESM1_ESM.docx]

**Supplementary information**

Table S1: Plasmids for expression of chemokines.

| Construct | Template (**amplified insert**) | 5' forward primer | 5' reverse primer | Restr. enzymes | |
| --- | --- | --- | --- | --- | --- |
| pSUMO hCCL19 | Published in [[36](#_ENREF_36)] | - | - | | - |
| pSUMO hCCL5 E66S | pDNR-Dual **hCCL5** (DNASU #HsCD00002557) | TTAACCAGTGGGTCTCAGGTGGTTCCCCATATTCCTCGGACACC | TTAACTCGAGTTATTACTAGCTCATCGACAAAGAGTTGATGTACTC | | BsaI, XhoI |
| pSUMO hCXCL12 | pDONR221 **hCXCL12** (DNASU #HsCD00822029) | AATTCCAGTGGGTCTCAGGTGGTAAGCCCGTCAGCCTGAGC | TTAACTCGAGTTATTAGTTTAAAGCTTTCTCC | | BsaI, XhoI |
| pSUMO hCCL19-S6 | Published in [[36](#_ENREF_36)] | - | - | | - |

Table S2: Plasmids for expression of single proteins.

| Construct | Template (**amplified insert**) | 5' forward primer | 5' reverse primer | Restr. enzymes | |
| --- | --- | --- | --- | --- | --- |
| pcDNA3 ACKR4-EGFP | Published in [[40](#_ENREF_40)] | - | - | | - |
| pcDNA3 ACKR4 | pcDNA3 **ACKR4**-EGFP | GGAGACCCAAGCTTCATTACGGGTACCCGTCATGGCTTTGGAACAAAATC | CCTCCGCTCTCGAGTCCACCGCGGCCGCTCATCAAATAGAGAAGGTAGAAGTAGGTTCAG | | KpnI, NotI |
| pcDNA3 S6-ACKR4 | pcDNA3 **ACKR4** | AATTGCGGCCGCGCCACCATGGGCGATAGCCTAAGTTGGCTGCTACGCCTGCTGAATAGCGGCGGTGGAGGATCCGCAGCTTTGGAACAAAATCAATC | GCGAGCTCTAGCATTTAGGTG | | NotI, XhoI |
| pcDNA3 HA-ACKR4 | pcDNA3 **ACKR4** | AATTAAGCTTGCCACCATGTACCCATACGACGTCCCAGACTACGCTAGCGGCGGTGGAGGATCCGCAGCTTTGGAACAAAATCAATCTACC | GCGAGCTCTAGCATTTAGGTG | | HindIII, XhoI |
| pcDNA3 ACKR4-HA | Published in [[26](#_ENREF_26)] | - | - | | - |
| pcDNA3 ACKR4-FLAG | pcDNA3 **ACKR4**-EGFP | CGAAATTAATACGACTCACTATAGGGAGACCC | GCTCCTCGCCCTTGCTCACTCTAGACTACTTATCGTCGTCATCCTTGTAATCGCTACCGCCACCGCCGGAACC | | HindIII, XbaI |
| pcDNA3 lgBiT-CAAX | pcDNA3 **lgBiT**-mGα_i_ [[49](#_ENREF_49)] | GGTAAAGCTAGCATGGTCTTCACACTCG | GGAACTGGATCCGTTGATGGTTACTCGGAAC | | NheI, EcoRI |
| pcDNA3 smBiT-βarrestin1 | pcDNA3 **βarrestin1**-Nluc [[26](#_ENREF_26)] | GAGACCCAAGCTTCGTCATGGTGACCGGCTACCGGCTGTTCGAGGAGATTCTGGGAGGTGGCGGTTCTGGTGGTGGCGGTTCCGGCGGTGGCGGTAGCGGCGACAAAGGGACCCG | CCAATCGATCCACCTCTAGATCATCATCTGTTGTTGAGCTGTG | | HindIII, XbaI |
| pcDNA3 smBiT-βarrestin2 | pcDNA3 **βarrestin2**-Nluc [[26](#_ENREF_26)] | AATTAAGCTTCGTCATGGTGACCGGCTACCGGCTGTTCGAGGAGATTCTGGGAGGTGGCGGTTCTGGTGGTGGCGGTTCCGGCGGT | GCGAGCTCTAGCATTTAGGTG | | HindIII, XbaI |
| pcDNA3 ACKR1 | pCMV6-XL5 **ACKR1** (Origene plasmid #SC125243) | AATTAAGCTTCATTACGATGGGGAACTGTCTGCA | TTAATCTAGACTAGGATTTGCTTCCAAGGG | | HindIII, XbaI |
| pcDNA3 ACKR1-HA | pCMV6-XL5 **ACKR1** (Origene plasmid #SC125243 | AATTAAGCTTCATTACGATGGGGAACTGTCTGCA | TTAACTCGAGGGATTTGCTTCCAAGGG | | HindIII, XhoI |
| pcDNA3 ACKR2 | Gift from Bernhard Moser | - | - | | - |
| pcDNA3 ACKR2-HA | pcDNA3 **ACKR2** | CGAAATTAATACGACTCACTATAGGGAGACCC | TTAACTCGAGGGCTGATTTATTCCCCACATCC | | HindIII, XhoI |
| pcDNA3 ACKR3 | Gift from Marcus Thelen | - |  | | - |
| pcDNA3 ACKR3-HA | pcDNA3 **ACKR3** | CGAAATTAATACGACTCACTATAGGGAGACCC | TTAACTCGAGTTTGGTGCTCTGCTCCAAGG | | HindIII, XhoI |
| pcDNA3 EYFP | pcDNA3 ACKR4-**EYFP** Published in [[26](#_ENREF_26)] | CGAAATTAATACGACTCACTATAGGGAGACCC | GCGAGCTCTAGCATTTAGGTG | | HindIII, BamHI |

Table S3: Plasmids for simultaneous expression of a fluorophore and a receptor.

| Construct | Template (**amplified insert**) | 5' forward primer | 5' reverse primer | Restr. enzymes | |
| --- | --- | --- | --- | --- | --- |
| pIRES mScarlet_ACKR4 | pcDNA3 **ACKR4** | ATATGGCGGCCGCAATGGCTTTGGAACAAAATCAATCTACC | GCGAGCTCTAGCATTTAGGTG | | NotI, XbaI |
| pIRES YPet_ACKR4-HA | pcDNA3 **ACKR4-HA** | ATATGGCGGCCGCAATGGCTTTGGAACAAAATCAATCTACC | GCGAGCTCTAGCATTTAGGTG | | NotI, XbaI |
| pIRES EYFP_ACKR4 | pcDNA3 **ACKR4** | ATATGGCGGCCGCAATGGCTTTGGAACAAAATCAATCTACC | GCGAGCTCTAGCATTTAGGTG | | NotI, XbaI |
| pIRES EYFP_ACKR4-HA | pcDNA3 **ACKR4-HA** | ATATGGCGGCCGCAATGGCTTTGGAACAAAATCAATCTACC | GCGAGCTCTAGCATTTAGGTG | | NotI, XbaI |
| pIRES YPet_ACKR4 F348A/I305A | pcDNA3 **ACKR4 F348A/I350A** | ATATGGCGGCCGCAATGGCTTTGGAACAAAATCAATCTACC | GCGAGCTCTAGCATTTAGGTG | | NotI, XbaI |
| pIRES EYFP_ACKR4 F348A | pcDNA3 **ACKR4 F348A** | ATATGGCGGCCGCAATGGCTTTGGAACAAAATCAATCTACC | GCGAGCTCTAGCATTTAGGTG | | NotI, XbaI |
| pIRES EYFP_ACKR4 I305A | pcDNA3 **ACKR4 I350A** | ATATGGCGGCCGCAATGGCTTTGGAACAAAATCAATCTACC | GCGAGCTCTAGCATTTAGGTG | | NotI, XbaI |
| pIRES EYFP_ACKR4 F348A/I305A | pcDNA3 **ACKR4 F348A/I350A** | ATATGGCGGCCGCAATGGCTTTGGAACAAAATCAATCTACC | GCGAGCTCTAGCATTTAGGTG | | NotI, XbaI |

Table S4: Primers used for site-directed mutagenesis.

| Construct | Template (**amplified insert**) | 5' forward primer | 5' reverse primer | Restr. enzymes | |
| --- | --- | --- | --- | --- | --- |
| pcDNA3 ACKR4 F348A | pcDNA3 **ACKR4** | CTGAACCTACTTCTACCGCCTCTATTTGATGAGCG | CGCTCATCAAATAGAGGCGGTAGAAGTAGGTTCAG | | - |
| pcDNA3 ACKR4 I350A | pcDNA3 **ACKR4** | CTTCTACCTTCTCTGCTTGATGAGCGGCCG | CGGCCGCTCATCAAGCAGAGAAGGTAGAAG | | - |
| pcDNA3 ACKR4 F348A/I305A | pcDNA3 **ACKR4** | CTGAACCTACTTCTACCGCCTCTGCTTGATGAGCG | CGCTCATCAAGCAGAGGCGGTAGAAGTAGGTTCAG | | - |


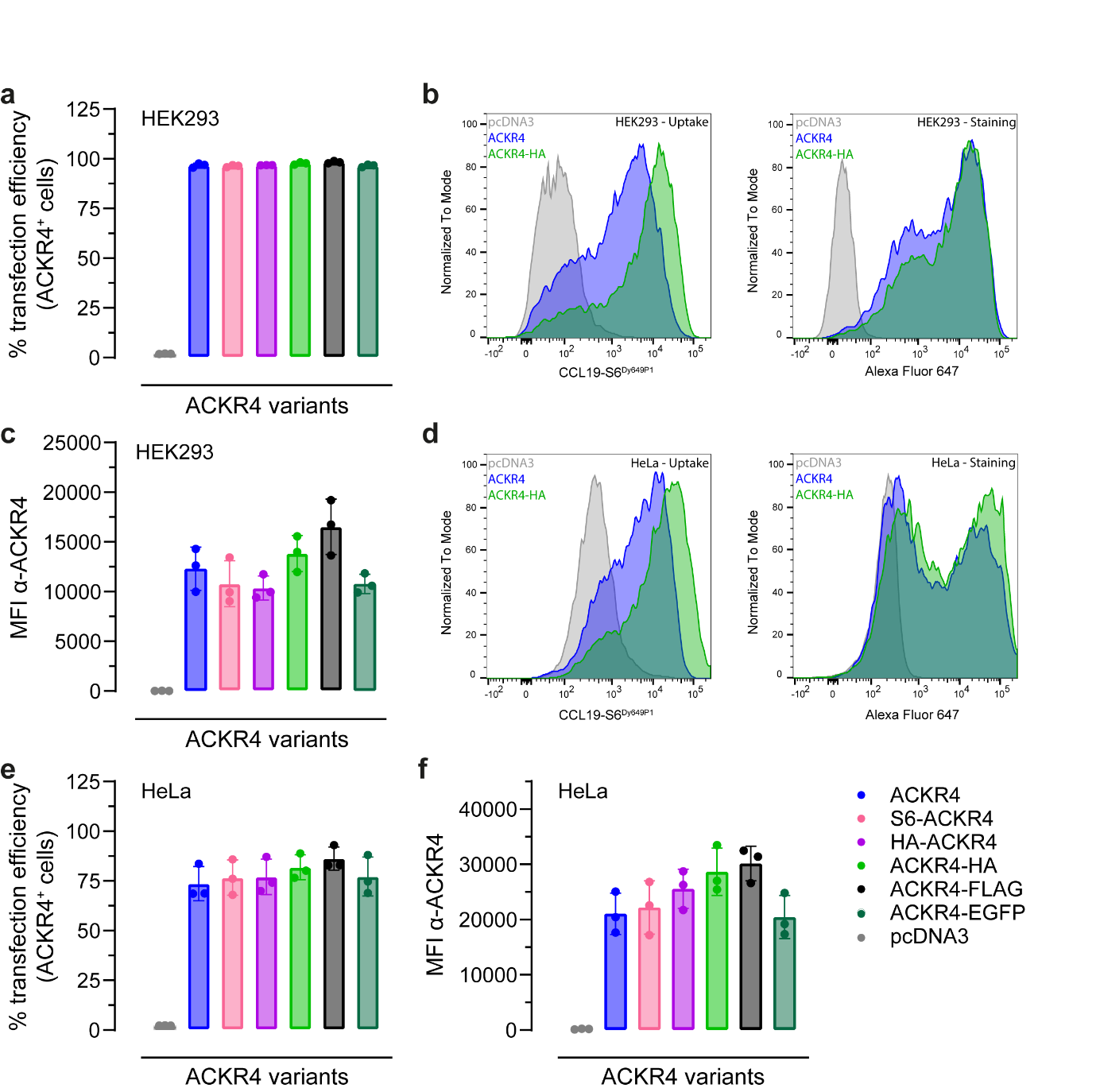


Figure S1: **Cell surface expression of native and tagged ACKR4 variants (related to Figure 1).** **a** Transient transfection efficiency in HEK293 cells. Percentage of HEK293 cells positive for ACKR4 surface expression after transfection with different ACKR4 constructs. n = 3, mean ± SD. **b** Representative experiment depicting CCL19-S6^Dy649P1^ chemokine uptake (one of three experiments shown in Figure 1a) and antibody-mediated surface staining of HEK293 cells transiently transfected with ACKR4 or ACKR4-HA as determined by flow cytometry. **c** Surface expression of indicated ACKR4 variants in transiently transfected HEK293 cells was determined by flow cytometry using an anti-ACKR4 antibody for staining. n = 3, mean ± SD. **d** Representative flow cytometry histograms of chemokine uptake (one of three experiments shown in Figure 1c) and antibody surface staining in transiently transfected HeLa cells expressing ACKR4 or ACKR4-HA. **e** Transient transfection efficiency in HeLa cells. Percentage of HeLa cells expressing the different ACKR4 variants at the cell surface. n = 3, mean ± SD. **f** Surface expression of indicated ACKR4 variants in transiently transfected HeLa cells was determined by flow cytometry using an antibody against ACKR4. n = 3, mean ± SD. MFI values in (c) and (f) were used for receptor normalization in Figure 1b and 1d, respectively.


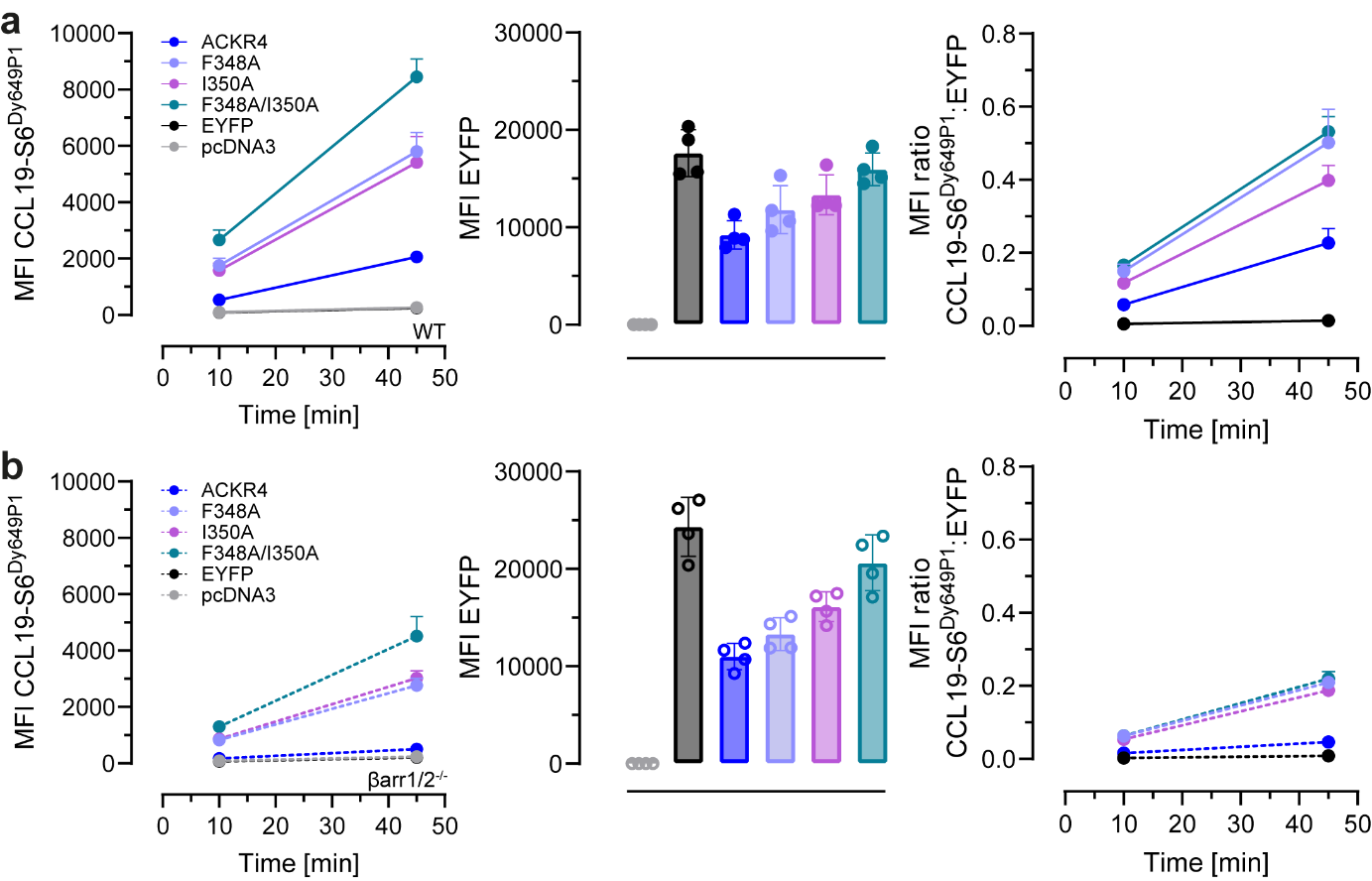


Figure S2: **Mutation of a putative class II PDZ-binding domain at the tip of ACKR4 enhances CCL19 internalization (Related to Figure 5).** HEK293 wild-type (WT; **a**) or βarrestin1/2^-/-^ (βarr1/2^-/-^; **b**) cells were transiently transfected with an empty vector, EYFP only or pIRES vectors encoding for EYFP and the indicated ACKR4 mutants, were stimulated with 5 nM CCL19-S6^Dy649P1^ and analyzed by flow cytometry. Chemokine uptake (left), EYFP expression (middle) and MFI ratio between CCL19-S6^Dy649P1^ and EYFP (right) is shown. n = 4, mean ± SD.
